# Dynamic and Robotic Computer‐Assisted Implant Surgery—A Possible Workflow for the Future?

**DOI:** 10.1111/adj.70013

**Published:** 2025-11-04

**Authors:** Yulan Wang, Ting Xia, Sebastian Kühl, Valentin Herber, Michael M. Bornstein

**Affiliations:** ^1^ State Key Laboratory of Oral & Maxillofacial Reconstruction and Regeneration, Key Laboratory of Oral Biomedicine Ministry of Education, Hubei Key Laboratory of Stomatology School & Hospital of Stomatology, Wuhan University Wuhan China; ^2^ Department of Research University Center for Dental Medicine Basel UZB, University of Basel Basel Switzerland; ^3^ Department of Oral Surgery University Center for Dental Medicine Basel UZB, University of Basel Basel Switzerland; ^4^ Department of Oral Health & Medicine University Center for Dental Medicine Basel UZB, University of Basel Basel Switzerland

**Keywords:** dental implant, dental robotics, digitalization, dimensional measurement accuracy, dynamic navigation

## Abstract

Digital technologies are reshaping dental implantology, with dynamic navigation and robotic systems offering high implant placement accuracy within clinically acceptable error ranges, the latter often achieving slightly higher accuracy. These systems enhance surgical accuracy and minimise trauma; however, high costs, extended preparation time, steep learning curves and uncertain patient acceptance limit their widespread adoption. This review summarises current principles, applications, benefits and limitations of dynamic navigation and robotic computer‐assisted implant surgery (d‐CAIS and r‐CAIS), highlighting the need for clinicians to refine system proficiency and adapt their roles for future implant treatment procedures.

**Summary**

- Advancements in dynamic and robotic computer‐assisted technologies are reshaping the accuracy and predictability of dental implant surgery.
- This review systematically summarises the recent principles, applications, advantages and limitations of dynamic navigation and robotic technologies in implantology as well as other dental fields.
- Understanding these systems and their clinical application is crucial for practitioners preparing for the digital future of implant dentistry.

## 1 Introduction

The application of digital technologies in the field of dentistry spans multiple areas and specialties, and has transformed many tasks from diagnosis to treatment, with digital methods gradually replacing traditional approaches [1]. Digital dental workflows primarily include obtaining patient image data (e.g., using cone beam computed tomography/CBCT, intraoral scanning and facial scanning), virtual patient and treatment planning, digital fabrication of materials used during interventions (such as digital titanium meshes, digital bone blocks) or patient treatment by means of digital tools (such as surgical guides, dynamic navigation) all with the goal of achieving more personalised, patient‐centered dental care [2]. The rapid advancement of these digital tools has not only enhanced the precision and safety of treatments and simplified clinical workflows, but also aims to reduce the likelihood of patient morbidity and complications.

In the field of oral implantology, digital technologies are also employed to enhance the precision of surgeries, particularly the accuracy of implant placement [3, 4]. These methods assist in achieving prosthetically driven implant positioning, which ensures optimal aesthetic and functional outcomes and long‐term implant stability. Additionally, they help in safeguarding critical anatomical structures from damage and significantly reduce the time of surgery. Computer‐assisted implant surgery (CAIS) involves virtual preoperative planning of implant positions based on collected patient data, and then translating this plan into reality within the patient's mouth [5]. Currently, this can be achieved through three methods: static CAIS (s‐CAIS), dynamic CAIS (d‐CAIS) and robotic CAIS (r‐CAIS) [1].

A s‐CAIS refers to a prefabricated surgical guide made of resin or metal that does not allow for flexible modifications during surgery. It is used to direct the preparation of the implant osteotomy and placement, achieving the digitally planned implant position [6, 7]. D‐CAIS, also known as dynamic navigation, is a real‐time system that guides the clinician during the procedure without the use of a physical guide, digitally realising the planned implant position by allowing observation and adjustment of the relative position between the implant drill and the patient's anatomical structures on a screen, smart glasses or other devices [7, 8]. R‐CAIS represents a significant advancement over d‐CAIS in surgical procedures by employing a robotic arm to replace the surgeon's hand to directly execute implant positioning as designed by the computer system [9, 10]. This approach reduces potential errors that may arise from the human‐screen coordination required in d‐CAIS. Additionally, it might improve safety by compensating for trembling or uncontrolled movement.

Although robotics have received widespread attention, they are not without limitations. For example, the application of dental robotics in complex clinical scenarios is limited due to technical constraints, and aspects such as economic efficiency and patient acceptance have not been fully explored. While the adoption of robotic technology has begun to reshape the role of traditional implant dentistry, it prompts a pivotal question: what role will dentists play in the future of oral implantation? The main objective of this paper is to introduce the principles, applications, advantages and limitations of dynamic navigation and robotic technologies in dental implantology. Furthermore, it explores their potential applications in improving patient treatment experiences and enhancing healthcare efficiency, as well as their potential future development.

## 2 Current State of Dynamic Computer‐Assisted Implant Surgery (d‐CAIS)

### 2.1 Development and Principles of d‐CAIS

There are several limitations of s‐CAIS, including the inability to modify treatment planning during surgery, inadequate water cooling, high requirements for patient mouth opening. Additionally, inaccuracies in the fabrication of surgical guides can affect their fit and stability. Possible positioning errors of the guide during surgery also remain a concern. To overcome these limitations [11, 12], d‐CAIS was proposed in the early 2000s and has since undergone gradual development [8].

D‐CAIS refers to using a dynamic navigation system that enables clinicians to monitor in real time the relative position between the handpiece, virtual implant plan and the patient's anatomical structures during surgery. These systems integrate preoperative data collected from the patient to reconstruct anatomical details, design virtual implant positioning and provide real‐time tracking of the handpiece and the patient throughout the procedure [8].

**FIGURE 1:**
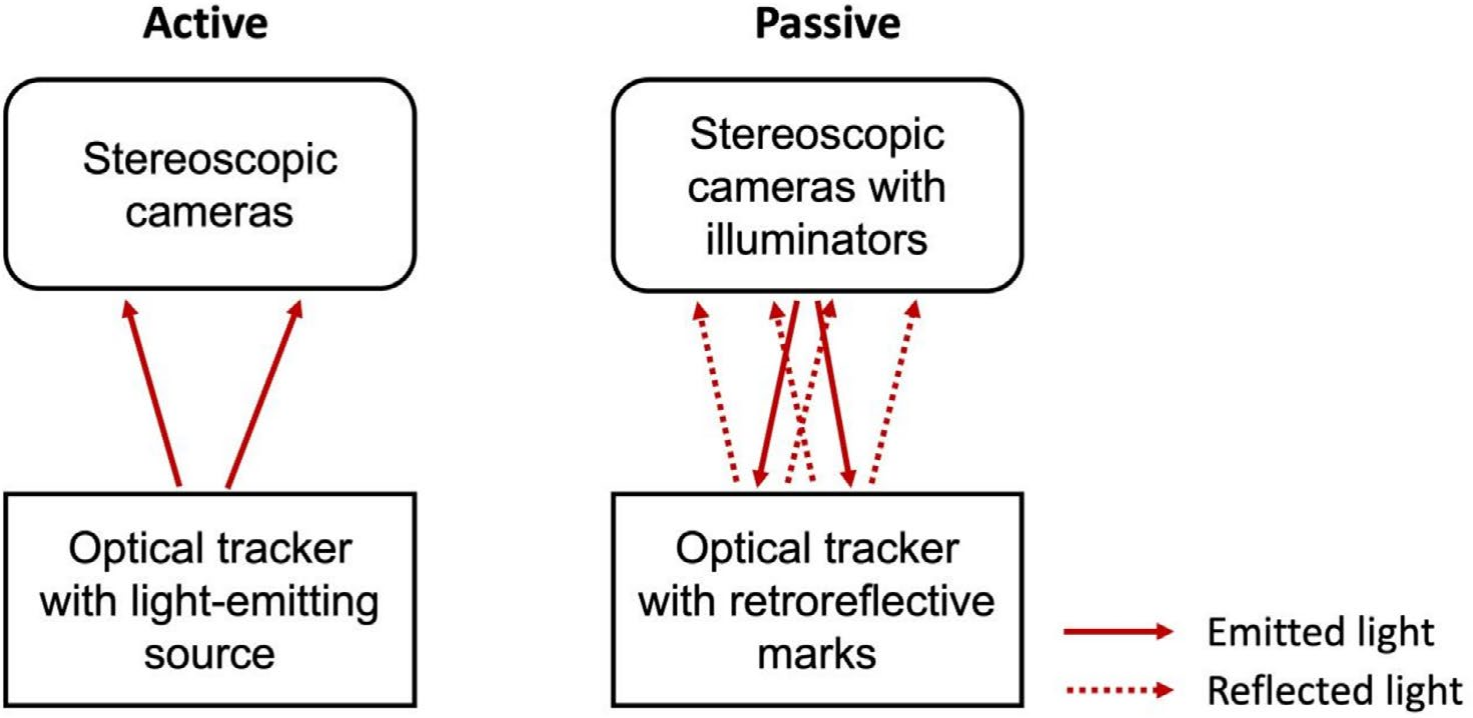
The different working principles of active (left) and passive (right) optical tracking.

There are two primary methods for tracking the movement of the handpiece or patient during surgery using optical dynamic navigation: active or passive optical tracking. Active optical tracking involves a light source on the optical tracker that actively emits light, which is then detected by a camera. In contrast, passive optical tracking uses an external light source (usually placed beside the camera) to emit light, which is reflected by the reflective markers on the optical tracker. The cameras then track the light reflected from these markers (shown in Figure 1) [13, 14]. Research has shown that the dynamic navigation system with active optical tracking demonstrated higher implant accuracy compared to the system with passive optical tracking [15].

To achieve real‐time guidance for dentists, there are currently two approaches to d‐CAIS. One method is the conventional navigation approach, where the dentist observes the patient's CBCT information, the virtual implant position and the current relative position of the handpiece and oral structure on a screen. The dentist then adjusts the handpiece's position in the patient's actual oral cavity to execute the preoperative plan. This method requires the dentist to separate hand and eye movements, demanding excellent coordination. Additionally, the act of switching focus from the screen to the actual patient disrupts the routine workflow, leading to longer procedure times, increased dentist fatigue and potentially raising the risk of adverse intraoperative outcomes. Finally, due to the missing physical depth stop, the risk of nerve injury is additionally increased.

Another approach for dentists is to use augmented reality (AR) and mixed reality (MR) devices for real‐time navigation, such as head‐mounted displays (HMDs) or integral videography (IV) overlay systems. This approach overlays calibrated instruments, planned implant paths and 3D anatomical reconstructions onto the surgical field, anchoring the virtual preoperative plan in real time to guide the procedure and reduce workflow disruptions, procedure time and operator fatigue [16, 17, 18]. Although the concept appears promising, AR/MR systems face significant technical, usability and adoption barriers, including latency [19], display constraints [20], complex workflows [21] and high costs [22].

#### 2.1.1 Composition of Dynamic Navigation System

Dynamic navigation is achieved through the coordination of the navigation system, optical tracking system and the surgeons' collaborative efforts. It consists of the following components.

##### 2.1.1.1 Real‐Time Navigation System

The system is responsible for running the planning and guidance software, integrating preoperative CBCT data, patient positioning and surgical instrument positioning, displaying this information in real time on a screen during the procedure.

The real‐time calculation requires additional computer hardware with high Central Processing Units (CPU).

##### 2.1.1.2 Optical Tracker

**FIGURE 2:**
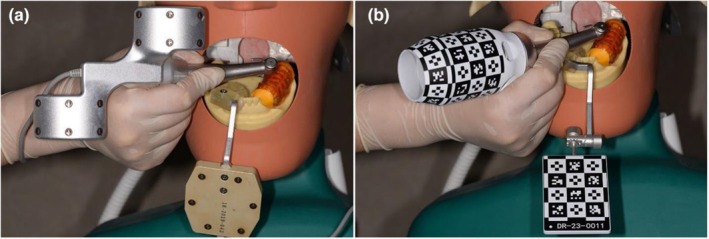
Image of optical tracker attached to the patient's jaw and the handpiece. Active (a) and passive (b) dynamic navigation approaches [23].

Includes one optical tracker rigidly attached to the patient's jaw and one optical tracker rigidly attached to the handpiece (Figure 2). They may actively emit light sources or passively reflect light emitted by an external source.

##### 2.1.1.3 Cameras

Typically using stereo cameras, it tracks the positions of the patient and the surgical instruments in real time throughout the procedure.

##### 2.1.1.4 AR or MR Devices

In AR or MR d‐CAIS, an additional device—such as glasses or head‐mounted displays—is used to provide guidance for dentists.

#### 2.1.2 Workflow of d‐CAIS

##### 2.1.2.1 Preoperative Patient Data Collection

Radiopaque fiducial markers are fixed in the patient's oral cavity prior to CBCT imaging. These intraoral markers must remain in place during the CBCT scan and are to be reused during surgery, ensuring they are positioned exactly as they were during imaging for accurate reproducibility. Alternatively, anatomical landmarks within the oral cavity can serve as markers instead of fiducial markers [8].

##### 2.1.2.2 Surgical Plan Design

The dentists determine the implant's position, angle and depth based on the patient's preoperative data and treatment plan [8].

##### 2.1.2.3 Registration and Calibration

Registration denotes a process that aligns the patient's anatomic landmarks to a corresponding 3D object in a cohesive coordinate in the navigation system [23]. Currently, there are two primary registration methods in d‐CAIS:

1. Feature Point‐Based Registration (F‐PBR): This method is also known as marker‐free registration, which relies on anatomical points and features of the teeth from preoperative CBCT scans. It achieves point‐to‐point matching without requiring additional equipment.
2. Marker Point‐Based Registration (M‐PBR): This method employs radiopaque fiducial markers for registration. Currently, there are two primary approaches for fabricating these markers. The first method involves positioning and securing a marker on the patient's teeth using thermoplastic material prior to the CBCT scan. The second method entails the virtual design and 3D printing of a custom marker tray tailored to the patient's dental anatomy. Once printed, the tray is fixed to the patient's teeth during the surgery, ensuring a personalised fit. The first method provides a simpler and more immediate solution, while the second allows for a more tailored approach, though it has shown to be less accurate due to the 3D printing process [24]. In cases of fully edentulous jaws or situations where it is difficult to secure conventional fiducial markers, it becomes necessary to use mini‐screws as fiducial markers by fixing them within the patient's alveolar bone [25].

**FIGURE 3:**
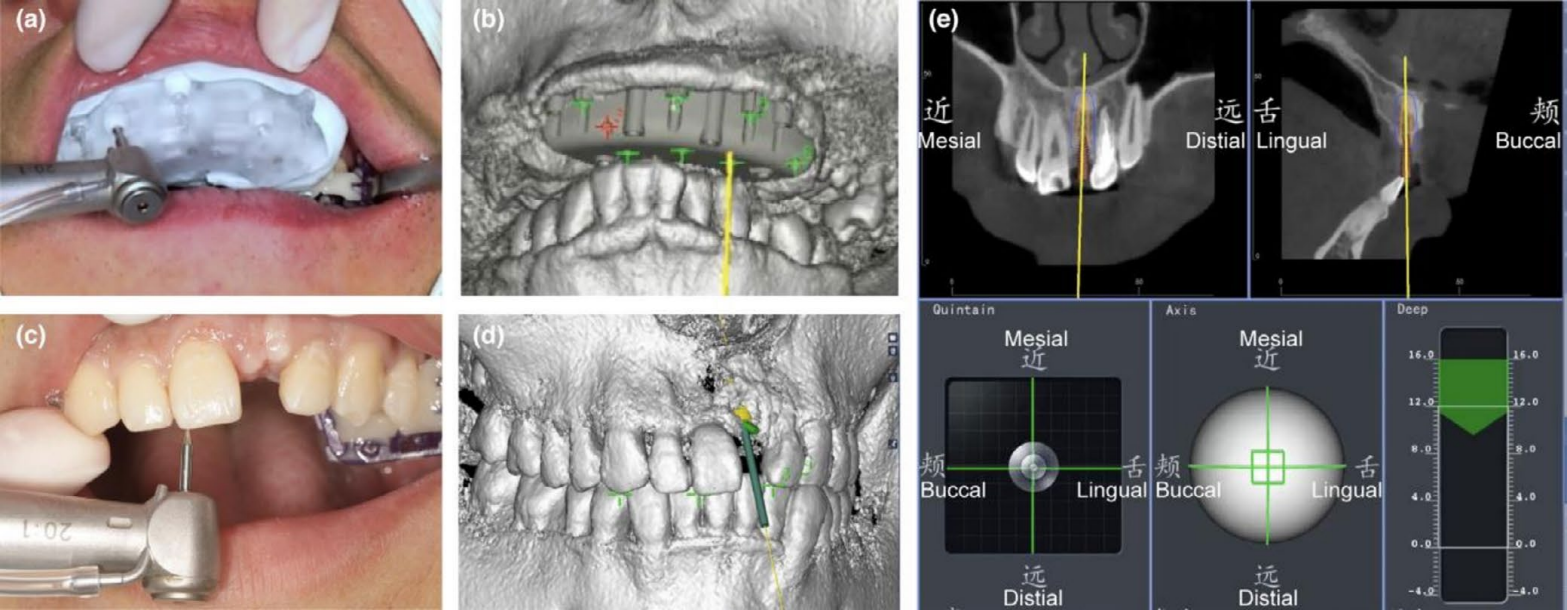
Surgical procedures for the M‐FBR and F‐PBR during d‐CAIS. The custom splint which was re‐positioned onto the patient's dental arch (a, b). The radiopaque fiducial markers on the splint were paired with the corresponding high‐density points shown in the CBCT to achieve marker‐based registration. F‐PBR was accomplished by pairing the selected cusps or fossae with the corresponding anatomic structures shown in the CBCT (c, d). The drilling and implant insertion procedures were guided by the navigation system (e) [27].

The underlying mechanisms of these two registration methods differ: F‐PBR is based on anatomical landmarks and features in CBCT scans, requiring no extra devices, whereas M‐PBR relies on the fiducial markers provided by the navigation system [24, 26]. Current research indicates that there is no significant difference in accuracy between these two methods. However, the F‐PBR approach does not need extra markers fixed in patients' mouths (Figure 3), requiring fewer clinical procedures and leading to higher postoperative satisfaction [27].

Prior to performing the surgery, it is essential to calibrate the surgical handpiece to ensure that the optical tracking system can provide accurate, real‐time feedback on the relative positions of the patient's anatomical structures and the drill position [8].

##### 2.1.2.4 Surgery

During the surgical procedure, the dynamic navigation system uses stereoscopic cameras to track the position of surgical instruments in real time and compares their location with the pre‐set targets and information. The system provides real‐time feedback, allowing the dentist to adjust the instrument's position based on the live information displayed on the screen, ensuring precise implant placement.

### 2.2 The Current Application of d‐CAIS in Dental Implantology

The application of d‐CAIS in dental implantology has become increasingly widespread. Currently, there are several commercially available systems or prototype devices on the market as shown in Table 1. d‐CAIS is now utilised in immediate implant placement, full‐arch implant surgeries, transcrestal sinus lift elevation (tSFE) and conventional implant procedures. The main advantages of using d‐CAIS are as follows.

**TABLE 1:** D‐CAIS clinical studies summarised by device.

Device	Patients/implants	Mean positional deviation (platform/apex/angle)	Study types and references	Indications	Tooth sites	Survival/follow‐up	Complications	Patient‐centered outcomes
Iris‐100	107/140	0.44–1.24 mm/0.49–1.58 mm/1.03°–3.78°	RCT [28], RCT [29], RCT [30], RCT [31]	Immediate; Healed; Single‐tooth; Partially edentulous	All sites	100% (range, 13–36 months)	Without any significant complications	NR
Dcarer	129/177	0.44–1.36 mm/0.85–1.48 mm/2.08°–4.28°	Pilot study [32], RCT [27], RCT [33], RCT [34], RCT [35], Retrospective study [36], Retrospective study [37]	Immediate; Early; Healed; Single‐tooth; Partially edentulous; TSFE	All sites	100% (where reported)	No complications	Good patient satisfaction, especially for marker free group
Navident	65/103	0.66–6.57 mm/1.00–3.77 mm/2.26°–8.89°	RCT [38], RCT [39], RCT [40], RCT [41], Retrospective study [42], Split mouth RCT [43]	Healed; Partially edentulous; Fully edentulous; Zygomatic implants; Pterygoid implants	All sites	97.5% (4 month after surgery); 100%; at impression taking; 100% (3‐mon after implantation)	No adverse events	Less early postoperative pain in flapless group; Complaining about longer surgery time in d‐CAIS group
VectorVision2	84/271	1.37–1.57 mm/1.99–2.10 mm/2.25°–2.68°	Pilot study [44], Retrospective study [45]	Fully edentulous; Zygomatic implants; Zygomatic implants	Maxilla	98.64% (24.11 month follow up)	28 devices related negative events, 2 ZI failures due to implant malposition	NR
X‐Guide	80/216	1.17–1.60 mm/1.30–1.83 mm/2.19°–3.80°	Prospective tiral [46], Retrospective study [47]	Immediate; Healed; Partially edentulous; Fully edentulous	All sites	NR	NR	NR
ImplaNav	10/18	1.04 mm; 1.35 mm; 6.46*°*	Prospective trial [48]	Partially edentulous; Fully edentulous	NR	NR	NR	NR
AR‐d‐CAIS (Iris–100 + AR glasses) (Epson Moverio BT‐300)	10/10	0.75 ± 0.45 mm; 0.87 ± 0.45 mm; 1.47° ± 1.01°	RCT [49]	Healed; Single‐tooth	All sites	NR	No major complications	NR

*Note:* Only d‐CAIS cohorts were counted (freehand, s‐CAIS and r‐CAIS controls excluded), patients/implants are cumulative across included robot studies for each system. Where multiple clinical studies were available for a given device, the lowest and highest reported mean values across all included studies were used to construct the range. This summary provides a descriptive overview of device‐specific performance under DN conditions. Indications include timing (immediate/healed/etc.), edentulism status and special sites (zygomatic, pterygoid, TSFE, bridge).

Abbreviation: NR, not reported.

#### 2.2.1 The Advantages of d‐CAIS in Dental Implantology

##### 2.2.1.1 High Positional Accuracy

Based on the summary presented in Table 1, it is evident that the current positional accuracy of d‐CAIS is high. At conventional single sites, platform, apex and angular deviations of d‐CAIS typically show mean deviations of ~0.44–1.07 mm (platform), 0.76–1.29 mm (apex) and 2.08°–4.28° (angular) across clinical studies. Variability is substantially higher at complex sites (e.g., zygomatic or pterygoid).

##### 2.2.1.2 Wide Applicability

Although there is no significant difference in accuracy between d‐CAIS and s‐CAIS, s‐CAIS requires greater mouth opening for the placement of the surgical template and longer drills, compared to d‐CAIS. In contrast, d‐CAIS can be operated with a common handpiece, meaning the requirement for mouth opening is no different from freehand procedures. In posterior regions with limited mouth opening, poor illumination hinders assessment of implant position by freehand technique; d‐CAIS overcomes this by providing on‐screen positional guidance. For complex implant surgeries, such as zygomatic or pterygoid implants, d‐CAIS markedly improves accuracy while greatly reducing the limitations imposed by mouth opening requirements when using s‐CAIS [38].

##### 2.2.1.3 Simplify the Treatment Process

In contrast to s‐CAIS, d‐CAIS allows for CBCT imaging, surgical planning and execution on the same day without waiting for template fabrication in the lab. Regarding surgical time, in complex procedures—such as pterygoid implant surgeries—using d‐CAIS significantly shortens the surgery time compared to freehand techniques [38]. This reduction is likely due to d‐CAIS saving the clinician time that would otherwise be spent making judgements during complex procedures [38].

##### 2.2.1.4 Reduce Postoperative Trauma

With the use of d‐CAIS, similar implant accuracy can be achieved using both flap and flapless surgeries, while patients experience reduced postoperative pain in the flapless group. This is particularly significant in cases such as zygomatic implant surgeries, which traditionally require extensive flap procedures. By minimising the need for large flap elevations, d‐CAIS greatly reduces both the surgical time required for flap management and the associated postoperative discomfort [50]. D‐CAIS is also used to precisely plan the location of the bone window for lateral access in maxillary sinus floor elevation procedures, which enables precise window opening, reducing complications and tissue injury, and may lessen postoperative trauma [51, 52].

##### 2.2.1.5 Effective Teaching Tool

D‐CAIS serves as an effective learning platform and is now gradually utilised to enhance students' precision and confidence in implant placement. Studies show that d‐CAIS is being incorporated into simulation‐based training for dental students, allowing students to practice implant placement with real‐time digital guidance to familiarise themselves with digital workflows and improve their technical skills before clinical application [53]. With repeated practice, proficiency gradually improves, enabling learners to achieve precise three‐dimensional positioning more rapidly than with traditional freehand methods [54, 55, 56].

#### 2.2.2 The Limitations of d‐CAIS in Dental Implantology

##### 2.2.2.1 Economic Burden

Currently, one of the complicating issues with d‐CAIS limiting its wide market penetration lies in the high initial investment required for the equipment [57]. It is evident that current research was predominantly carried out in university‐affiliated hospitals, with significantly fewer studies conducted in private practices. Additionally, operators must simultaneously monitor the patient's intraoral situation, and the real‐time information displayed on the computer screen, necessitating additional training for dentists [58]. For individuals with varying levels of experience in implant surgery, the learning curve for using d‐CAIS does not differ significantly. Consequently, even highly experienced dentists require additional training to effectively adopt this technology [47, 59].

##### 2.2.2.2 Extended Surgical Time

Although previous discussions have highlighted that d‐CAIS can save time in complex procedures, in routine surgeries—such as single‐tooth implant placements—d‐CAIS tends to require more time than freehand and s‐CAIS. This is because in complex cases, the reduced need for large flap elevations and related suturing significantly shortens the overall procedure, whereas in simple cases, the extra time spent on registration and calibration is not offset by these benefits. In one study, patients in the navigation group expressed complaints about the longer surgical duration associated with d‐CAIS [39].

##### 2.2.2.3 Systematic Errors

Data acquisition, implant planning and registration are critical steps in d‐CAIS, subject to various sources of error. For example, metallic restorations or implants within the patient's mouth can create artefacts that may obscure anatomical structures or reference markers, thereby compromising registration accuracy [60]. Additionally, any movement of optical markers during CBCT scanning, patient movement during calibration, displacement of optical trackers during surgery, incorrect calibration of the drill and imprecise drilling technique can all adversely affect the accuracy of d‐CAIS [45].

#### 2.2.3 D‐CAIS Using Augmented Reality (Direct) Application—A Look Into Current Developments

AR‐d‐CAIS extends conventional dynamic navigation systems by adding a display device that overlays virtual planning information onto the physical world [17]. Currently, the predominant approach reported is to use commercially available AR glasses, which connect to the navigation system via Wi‐Fi or Bluetooth, and employ some software to achieve seamless interface integration and real‐time data exchange [61, 62]. Moreover, some reports describe fully commercialised AR dynamic navigation systems with pre‐configured AR devices such as Falcon Dynamic System (Straumann, Basel, Switzerland), Innooral System (Innoimplant Ltd., Budapest, Hungary) or self‐made AR‐d‐CAIS prototype(s). Although AR‐d‐CAIS appears more appealing in theory, when the three‐dimensional virtual image generated by AR is overlaid onto the real oral structures, discrepancies often arise between the virtual image and the actual situation due to overlay or positional errors. In addition, common discomforts reported by dentists during initial or prolonged use include dizziness, eye strain, neck pain and excessive device weight [63]. Randomised controlled trials have shown that, although these systems may improve accuracy compared with freehand surgery, they offer no advantage over d‐CAIS [49]. Consequently, some respondents perceive limited clinical value, with applications being more relevant to medical training and patient education [21]. Ongoing hardware and software improvements are required to address these limitations and facilitate broader and more effective implementation.

Summary: d‐CAIS is currently used worldwide in daily practice and can cover many clinical scenarios, offering numerous advantages including high accuracy, reduced postoperative trauma and serving as an effective teaching tool. However, one must also be aware of its current limitations such as economic factors, a potential increase in surgical time needed, and the need for specialised training in order to correctly select its use based on clinical needs. Related research has mainly focused on accuracy, while reports on implant survival/success rates, complications, surgical time, patient‐centred outcomes and other factors are still limited, necessitating more extensive research.

## 3 Current State of Robotic Computer‐Assisted Implant Surgery (r‐CAIS)

For d‐CAIS—whether using conventional or AR approaches—the requirement that dentists use their own hands to carry out the virtual implant plan places significant demands on human–machine collaboration and results in a relatively steep learning curve. In addition, dentist fatigue may compromise accuracy. To address this challenge, r‐CAIS has emerged in recent years. Although its principles are similar to d‐CAIS, it uses a robotic arm to replace the surgeon's hands, providing stable mechanical control and force feedback. Throughout the procedure, the surgeon monitors each step and adjusts the surgical plan and drilling protocol as needed.

### 3.1 Composition of Dental Robots

Dental robotic systems typically comprise several core components.

#### 3.1.1 Robotic Control System

A computer program that manages and monitors the interaction between the robotic arm, the patient and other elements, ensuring precise execution at each stage of the operation.

#### 3.1.2 Imaging System

Integrates preoperative CBCT and implant path planning with real‐time visuals of the drill's position in the patient's mouth, enabling the dentist to closely track the procedure and make dynamic adjustments.

#### 3.1.3 Robotic Arm

A highly precise and dexterous arm capable of performing delicate operations within complex oral environments.

**FIGURE 4:**
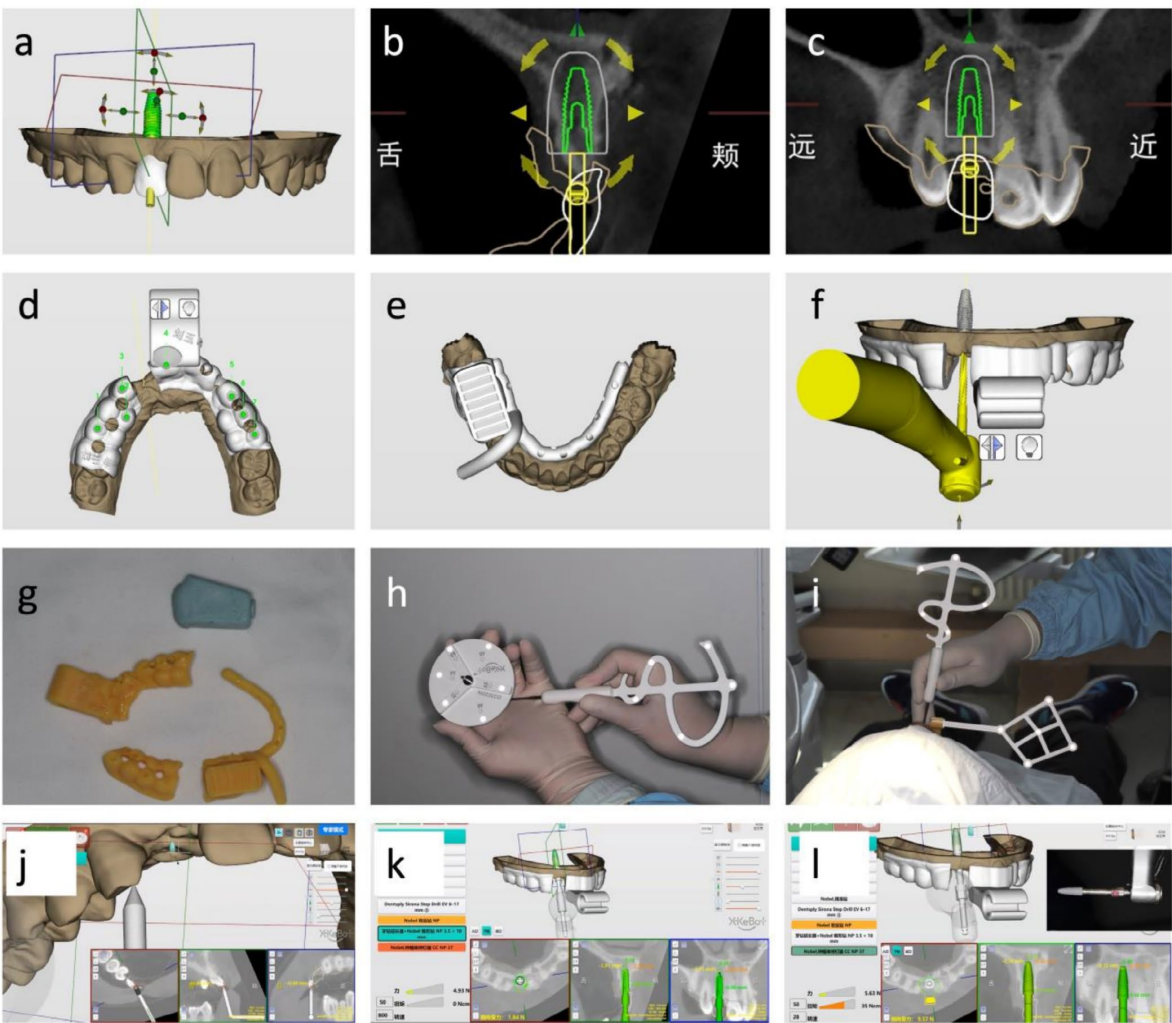
A case showing an active robotic workflow. Virtual implant planning (a–c); designing of surgical accessories for fixation of the optical tracker (d, e); planning of the route for the drill (f); 3D printed surgical accessories (g); registration process (h, i); calibration process (j); preparation of the implant bed (k); placement of the dental implant (l).

The workflow of r‐CAIS is similar to d‐CAIS, and its steps include data processing (including radiographic images and patient intraoral anatomical data), virtual implant planning, registration and calibration and surgical execution [64]. There is a gradual increase in the number of robotic systems available on the market, which are categoriSed into the following three types of robots based on their human‐machine interaction:

1. Active: This type of robot can actively enter and exit the patient's mouth on its own and can independently prepare the implant site and place the implant (e.g., YekeBot robot system is considered active) (Figure 4). During the procedure, the surgeon's primary tasks are to change the drills and monitor the robot's operation.
2. Semi‐active: This type of robot requires the surgeon to guide the robotic arm in and out of the patient's mouth, but the arm can independently drill and place the implant (e.g., Remebot and THETA robot system is considered active).
3. Passive: This type of robot requires the surgeon to guide the robotic arm into the mouth and also to guide the arm for both site preparation and implant placement. The main function of the robotic arm is to provide three‐dimensional positional guidance as well as control the depth of the drills and implants (e.g., Yomi robot system is considered active).

### 3.2 Advantages and Limitations

Clinical evidence demonstrates that r‐CAIS is highly safe, with virtually no intraoperative or postoperative adverse events or complications reported. The main potential failure modes are inaccurate registration/calibration or occlusion of the tracking markers; however, these issues can generally be resolved intraoperatively. Only one case has been reported in which a marker became loose during surgery, but it was successfully detected by the robotic system [65].

#### 3.2.1 Advantages

##### 3.2.1.1 High Positional Accuracy

Current evidence suggests that r‐CAIS can achieve high implant precision in both fully and partially edentulous patients. As shown in Table 2, based on currently published studies, the positional accuracy of robotic‐assisted implant placement systems demonstrates mean deviations ranging from 0.54 to 1.10 mm at the platform level, 0.54 to 1.50 mm at the apex, and 0.79° to 4.70° in angular deviation. Although the number of studies is still limited, evidence from RCTs and reviews indicates that r‐CAIS achieves the greatest implant positional accuracy—at the platform, apex and angular levels—compared with all other CAIS techniques [72, 74, 75].

**TABLE 2:** R‐CAIS clinical studies summarised by device.

Robotic system	Patients/implants (robot only)	Mean Robot positional deviation (platform/apex/angle)	Surgical time (robot)	Study types and references	Indications	Tooth sites	Complications	Patient‐centered/outcomes
THETA	90/90	0.76 mm/0.85 mm/2.05°	4.89–18.80 min	RCT [65, 66, 67]	Healed; Single‐tooth	All sites	NR	Implant failure reported
Remebot	20/69	0.67–0.74 mm/0.67–0.74 mm/1.11°–1.27°	NR	Case series [68, 69]	Immediate; Healed; Single‐tooth; Fully edentulous	NR	No complications reported	NR
Langyue collaborative robot	21/28	0.54 mm/0.54 mm/0.79°	20.00–70.00 min	Case series [70]	Single‐tooth; Fully edentulous	NR	No complications reported	NR
YakeRobot RS	28/35	0.65–1.10 mm/0.66–1.50 mm/1.52°–4.70°	1.80–21.00 min	Pilot Clinical study [71], Randomised controlled trial [72]	Healed; Single‐tooth; Partially edentulous	All sites	Large drill deviation noted; No complications reported	More pain reported compared with s‐CAIS l [72]
Yomi Robot	7/10	Fully guided (*n* = 7): 1.31 mm, 1.11 mm, 2.34. Partially guided (*n* = 3): 1.31 mm, 1.74 mm, 3.75°	NR	Prospective trial [73]	Healed; Partially edentulous	All sites	Buccal displacement observed	NR

*Note:* Only r‐CAIS cohorts were counted (freehand, s‐CAIS and d‐CAIS controls excluded). Patients/implants are cumulative across included robot studies for each system. Robot positional deviations are summarised as min–max of reported platform/apex/angular means. Indications list timing/status/special types; Tooth sites summarise anatomical coverage (‘All sites’ when full mouth).

##### 3.2.1.2 Shorter Learning Curve

Since the dentists' primary role during r‐CAIS is to assist and supervise the robot, a shorter learning curve is required, effectively diminishing the gap between novice and experienced surgeons. Studies indicate that stable implant placement accuracy can be achieved after only two or three practice sessions operating the robot [76]. Additionally, the robotic arm is not susceptible to problems of the human hand, including tremors due to fatigue or other psychological or physical factors.

#### 3.2.2 Limitations

Dental robots face similar limitations as d‐CAIS in terms of economic burden and system errors. Additionally, they have unique limitations.

Application is limited by tooth site and mouth opening. Because robotic arms are less flexible than the human hand and most systems restrict angulation along predefined paths, manoeuvring in narrow posterior regions—especially with limited mouth opening or anatomic constraints—remains challenging [70, 77].

##### 3.2.2.1 Operation Time

There is no consensus at present on the operation time required by r‐CAIS, primarily because different robotic systems vary in their design and require different operating procedures. Generally, it is believed that r‐CAIS may take a longer time than freehand or s‐CAIS, due to the additional preoperative preparation, calibration and registration required [78]. However, not all robotic systems are the same. Active robots, for instance, demand more extensive planning—such as determining the drill's spatial orientation, entry and exit paths and calibration or registration—resulting in the longest preparation times. In contrast, some semi‐automated robots feature automatic calibration and registration, which reduces preoperative preparation time. Likewise, research has shown that active and semi‐ active robots often require longer operative time than passive robots [77].

##### 3.2.2.2 Summary

r‐CAIS delivers high implant placement accuracy, but adoption is limited by cost and workflow. Systems are currently expensive and often more time‐consuming due to setup. Access to narrow posterior sites or in patients with limited mouth opening can be difficult.

## 4 Future Outlook and Developments

### 4.1 Applications of Robots in Other Fields of Dentistry

The application of dynamic navigation and dental robots is also actively applied to other fields of dentistry including endodontics, extraction of retained teeth, orthodontics, et al. They primarily use preoperative CBCT for precise target localization. Dental robots are useful in complex endodontic cases to ensure effective canal cleaning, shaping and accurate positioning, thereby enhancing surgical precision and efficiency, reducing risks of perforation, guidance errors and canal deviation caused by hand tremors or improper handling [79]. In apical surgery, dynamic navigation and robots were utilised for precise osteotomy for access and root resection, minimising damage to surrounding tissues and improving healing outcomes [80]. The ability of precise osteotomy is also applied to prepare a bony window for impacted teeth extraction [81] and lateral sinus floor elevation [51, 82]. In orthodontic treatment, dental robots are utilised in automated archwire bending based on preoperative digital data, thereby reducing human error, shortening treatment time and improving patient comfort [83].

### 4.2 Patient Trust in Dental Robots and Dynamic Navigations Including Economical Aspects

Contrary to initial concerns among dentists that patients would hesitate about robot‐assisted implant surgery [44], current evidence suggests the opposite: in a recent RCT, patient‐reported outcomes were comparable across s‐CAIS, d‐CAIS and r‐CAIS [72]. Another study reported significantly better patient‐centered domains (comfort, fear/anxiety‐related measures) with d‐CAIS compared with the freehand technique [84]. However, reporting on patients' perceptions is limited, highlighting the need for more research.

### 4.3 Future Trends in Robotic Developments in Oral Implantology

Despite current research demonstrating that r‐CAIS can improve positional accuracy, various limitations still persist. To further integrate these systems into clinical practice, future developmental directions may include the following.

#### 4.3.1 Expansion of Clinical Applications

Presently, robotic systems primarily focus on enhancing the positional accuracy of implant placement, resulting in relatively limited clinical scenarios. However, the force feedback capability of the robotic arm could be further exploited in other procedures—such as maxillary sinus floor elevation—by developing specialised instruments and integrating force feedback technology. This would expand the range of applications and improve the safety of additional clinical interventions.

#### 4.3.2 Enhancement of Robotic Arm Flexibility

Current limitations in the flexibility of the robotic arm necessitate a wide mouth opening to follow the preset access path. Future advancements that increase the arm's flexibility would enable the system to accommodate a broader range of patient anatomies. Technical improvements, such as more versatile angle adjustment mechanisms or a reduction in device size, are also recommended to broaden their application.

#### 4.3.3 Integration of Artificial Intelligence (AI) in Treatment Planning and the Changing Role of Dentists

Although still in an exploratory stage, AI in implantology already demonstrates practical value in assisting implant planning, guiding surgical template design and implant recognition based on radiographic or/and intraoral scanning data—yet its role remains supportive rather than decisive [85, 86, 87].

Looking ahead, AI is expected to advance toward autonomous functions. By leveraging large, multi‐modal datasets, future systems may enable truly personalised implant planning and execution through integration with robotics. Predictive algorithms can also be developed to forecast long‐term outcomes, such as implant survival and complication risks, strengthening follow‐up and maintenance. Yet, ethical, regulatory and data governance challenges remain key barriers to clinical adoption.

In this evolution, dentists' roles may shift toward individualised treatment planning, demonstrating surgical strategies and maintaining continuous patient interaction—aligned with the 4P principles of predictive, preventive, personalised and participatory care. While robotics enhance precision, surgeons remain indispensable for complex tasks such as flap design, suturing and tissue augmentation. This shift not only optimises outcomes but also allows clinicians to prioritise participatory care, including clear communication and comprehensive postoperative support.

### 4.4 Take‐Home Message for Dental Practice

Both d‐CAIS and r‐CAIS offer high accuracy. Routine sites show minimal platform/apex error and angular deviation, while complex sites (e.g., zygomatic/pterygoid) exhibit more variability.

#### 4.4.1 When to Choose What

Agility, versatility, cost control → d‐CAIS.

Mechanical control, repeatability → r‐CAIS.

#### 4.4.2 Safety/Complications

Serious device‐related events are rare. Errors typically arise from registration, fixation stability and workflow lapses—checklists can help.

#### 4.4.3 Patient Experience

RCT data show similar patient‐reported outcomes (PROs) for r‐CAIS, d‐CAIS and s‐CAIS, although PRO reporting remains limited.

#### 4.4.4 What Still Needs Evidence

Trials at complex sites, mid‐/long‐term outcomes (survival/MBL), cost‐effectiveness and more robust patient‐reported outcomes.

#### 4.4.5 Near‐Term Development Possibility

Marker‐free workflows, better ergonomics, increased angulation freedom, AR overlays and integrated quality assurance for registration and drill offsets.

## Disclosure

Permission to Reproduce Material: Figures 2 and 3 are reproduced with permission from John Wiley and Sons with proper citation.

## Conflicts of Interest

The authors declare no conflicts of interest.

## Data Availability

The authors have nothing to report.
